# Effects of supplemental fish oil on resting metabolic rate, body composition, and salivary cortisol in healthy adults

**DOI:** 10.1186/1550-2783-7-31

**Published:** 2010-10-08

**Authors:** Eric E Noreen, Michael J Sass, Megan L Crowe, Vanessa A Pabon, Josef Brandauer, Lindsay K Averill

**Affiliations:** 1 Department of Health Sciences, Gettysburg College, Gettysburg Pennsylvania, USA

## Abstract

**Background:**

To determine the effects of supplemental fish oil (FO) on resting metabolic rate (RMR), body composition, and cortisol production in healthy adults.

**Methods:**

A total of 44 men and women (34 ± 13y, mean+SD) participated in the study. All testing was performed first thing in the morning following an overnight fast. Baseline measurements of RMR were measured using indirect calorimetry using a facemask, and body composition was measured using air displacement plethysmography. Saliva was collected via passive drool and analyzed for cortisol concentration using ELISA. Following baseline testing, subjects were randomly assigned in a double blind manner to one of two groups: 4 g/d of Safflower Oil (SO); or 4 g/d of FO supplying 1,600 mg/d eicosapentaenoic acid (EPA) and 800 mg/d docosahexaenoic acid (DHA). All tests were repeated following 6 wk of treatment. Pre to post differences were analyzed using a treatment X time repeated measures ANOVA, and correlations were analyzed using Pearson's r.

**Results:**

Compared to the SO group, there was a significant increase in fat free mass following treatment with FO (FO = +0.5 ± 0.5 kg, SO = -0.1 ± 1.2 kg, p = 0.03), a significant reduction in fat mass (FO = -0.5 ± 1.3 kg, SO = +0.2 ± 1.2 kg, p = 0.04), and a tendency for a decrease in body fat percentage (FO = -0.4 ± 1.3% body fat, SO = +0. 3 ± 1.5% body fat, p = 0.08). No significant differences were observed for body mass (FO = 0.0 ± 0.9 kg, SO = +0.2 ± 0.8 kg), RMR (FO = +17 ± 260 kcal, SO = -62 ± 184 kcal) or respiratory exchange ratio (FO = -0.02 ± 0.09, SO = +0.02 ± 0.05). There was a tendency for salivary cortisol to decrease in the FO group (FO = -0.064 ± 0.142 μg/dL, SO = +0.016 ± 0.272 μg/dL, p = 0.11). There was a significant correlation in the FO group between change in cortisol and change in fat free mass (r = -0.504, p = 0.02) and fat mass (r = 0.661, p = 0.001).

**Conclusion:**

6 wk of supplementation with FO significantly increased lean mass and decreased fat mass. These changes were significantly correlated with a reduction in salivary cortisol following FO treatment.

## Background

It is generally believed that a high-fat diet is a contributing factor to excess body fat accumulation due to the greater energy density of fat and the relative inability of the body to increase fat oxidation in the presence of over consumption of fats [1,2]. However, several rodent studies have shown clearly that diets rich in omega 3 fatty acids, specifically eicosapentaenoic acid (EPA) and docosahexaenoic acid (DHA), which are found in large amounts in the oil from cold-water fish, lead to significantly lower total body fat stores vs diets rich in other fatty acids [3-7]. The exact mechanism(s) responsible for this phenomenon are not completely understood, but there are several possible explanations. For example, EPA and DHA are very effective at suppressing lipogenic gene expression [8,9], thereby limiting the synthesis of lipids. EPA and DHA have also been found to increase the oxidation of lipids as a result of an increase in carnitine acyltransferase I (CAT 1) activity [10,11], which allows greater fatty acid transport across the inner mitochondrial matrix via the carnitine-acylcarnitine translocase mechanism [12]. Additionally, EPA can increase mitochondrial lipid oxidation indirectly by inhibiting acetyl-CoA carboxylase [13], which is the enzyme that catalyzes the synthesis of malonyl CoA, and is a potent inhibitor of CAT I [14]. Moreover, EPA and DHA can also decrease the sensitivity of CAT I to malonyl CoA [11,15] which may allow a higher rate of lipid oxidation across a variety of different metabolic states. It is also possible that omega 3 fatty acids may influence total body lipid accretion by increasing thermogenesis as a result of increased activity of uncoupling proteins and peroxisomes [16], and/or by increasing lean body mass [3,5], which would indirectly increase thermogenesis.

Although there is some disagreement in the literature, there appears to be a negative effect of the stress hormone cortisol on body composition [17,18]. The well-documented association between Cushing's disease and obesity [19] clearly shows that conditions that significantly increase cortisol levels can increase fat accretion. However, it is not known if treatments that lower cortisol levels can positively impact body composition. There is limited evidence that fish oil supplementation can reduce cortisol levels [20], which raises the possibility that the consumption of fish oil could decrease body fat % by decreasing cortisol levels. To date, no study has examined the relationship between salivary cortisol and body composition following treatment with fish oil.

Despite the mechanistic data and results in rodents, very little is known about the effects of omega 3 fatty acids on body composition and metabolic rate in humans. In the first study using humans, Couet et al. [21] found that when 6 g/d of visible dietary fat was replaced with 6 g/d of fish oil for 3 wk, there was a significant increase in fat oxidation as measured by RER, and a concomitant decrease in total body fat as measured by dual energy X-ray absorptiometry. There was also an increase in the resting metabolic rate, but this was no longer evident when the observed slight increase in lean mass during the fish oil treatment was accounted for, perhaps suggesting that fish oil may increase RMR by increasing lean mass. More recently, Hill et al. [22] found that supplementing the diet with fish oil significantly reduced fat mass compared to a control group supplemented with sunflower oil. Similarly, Thorsdottir et al. [23] found that including fish, or fish oil supplements, in a hypoenergetic diet resulted in greater weight loss in young overweight men compared to a hypoenergetic diet that did not include fish or fish oil.

The aim of the present study was 1) to determine the effects of supplemental fish oil on body composition and resting metabolic rate in healthy adults, and 2) to determine the effects of supplemental fish oil on morning salivary cortisol concentrations, and determine if there is a relationship between changes in salivary cortisol concentrations and changes in body composition following fish oil treatment.

## Methods

Prior to all testing, approval for the study was obtained from the institutional review board at Gettysburg College and written informed consent was obtained from all subjects.

Healthy adults (18-55y) were recruited through flyers posted at Gettysburg College and surrounding community. Individuals who ate fatty fish at least 3 times a month, or were supplementing their diet with omega 3 fatty acids, or had a known metabolic or endocrine disorder were excluded. Subjects were healthy and active, but not engaged in consistent, systematic exercise training. In total, 44 individuals volunteered to participate (Table 1). Subjects were asked to maintain their current diet and exercise practices throughout the study.

**Table 1:** Pre and Post values following 6 weeks of treatment with 4 g/d of safflower oil, or 4 g/d of fish oil

	Safflower Oil			Fish Oil		
	**Pre**	**Post**	**Post-Pre** **Difference**	**Pre**	**Post**	**Post-Pre** **Difference**

Sex						
Male (n)	8			6		
Female (n)	14			16		

Age (y)	35 ± 14y (29;41)			33 ± 13y (27;39)		

Weight (kg)	71.1 ± 15.2 (64.7;77.5)	71.3 ± 15.3 (65.1;77.6)	0.2 ± 0.8 (-0.2;0.6)	71.3 ± 14.4 (65.1;77.6)	71.3 ± 13.7 (65.1;77.6)	0.0 ± 0.9 (-0.4;0.4)

Body Fat (%)	27.7 ± 10.6 (23.0;32.4)	28.0 ± 10.8 (23.2;32.8)	0.3 ± 1.5† (-0.4;1.0)	30.5 ± 7.7 (26.7;32.5)	30.1 ± 7.6 (26.3;33.9)	-0.4 ± 1.3† (-1.2;0.2)

Fat Mass (kg)	19.7 ± 9.7 (15.4;24.0)	19.9 ± 9.9 (15.5;24.3)	0.2 ± 1.2* (-0.3;0.7)	22.3 ± 8.2 (18.3;25.7)	21.8 ± 7.6 (18.2;25.0)	-0.5 ± 1.3* (-1.1;0.1)

Fat Free Mass (kg)	50.5 ± 11.9 (45.2;55.5)	50.4 ± 12.3 (45.0;55.8)	-0.1 ± 1.2** (-0.6;0.4)	50.1 ± 11.7 (45.1;55.1)	50.6 ± 11.9 (45.5;55.6)	0.5 ± 0.5** (0.3;0.8)

Salivary Cortisol (μg/dL)	0.305 ± 0.240 (0.212;0.399)	0.321 ± 0.311 (0.217;0.425)	0.016 ± 0.272 (-0.108;0.140)	0.270 ± 0.179 (0.179;0.361)	0.206 ± 0.131 (0.104;0.308)	-0.064 ± 0.142 (-0.127;-0.002)

RMR (24 h Kcal); n = 26	1290 ± 295 (1103;1477)	1228 ± 277 (1053;1400)	-62 ± 184 (-179;55)	1335 ± 213 (1200;1470)	1352 ± 323 (1147;1557)	17 ± 260 (-148;152)

RER; n = 26	0.809 ± 0.052 (0.776;0.842)	0.832 ± 0.41 (0.806;0.858)	0.023 ± 0.54 (-0.011;0.057)	0.841 ± 0.59 (0.804;0878)	0.822 ± 0.48 (0.791;0.853)	-0.019 ± 0.85 (-0.073;0.035)

Data are expressed as means ± SD (95% confidence interval). Data were analyzed using a treatment X time repeated measures ANOVA

* significant treatment X time interaction, p = 0.04

** significant treatment X time interaction, p = 0.03

† treatment X time interaction, p = 0.08

### Experimental Protocol

Subjects reported to the laboratory first thing in the morning following a 10-12 h overnight fast for RMR determination using open circuit indirect calorimetry (n = 26) and body composition assessment using air displacement via the Bod Pod^® ^(n = 44). Following these tests, a saliva sample was taken via passive drool and later analyzed for cortisol content. Subjects were then randomly assigned in a double blind manner to one of two groups:

***Safflower oil (SO)***: 4 g/d of safflower oil (Genuine Health Corporation, Toronto, Ontario, CA) administered in 4 enteric-coated capsules (each capsule provided 1 g of cold pressed, high linoleic acid, safflower oil).

***Fish oil (FO)***: 4 g/d concentrated fish oil (o3mega extra strength, Genuine Health Corporation, Toronto, Ontario, CA) administered in 4 enteric-coated capsules (each capsule provided 400 mg EPA and 200 mg DHA).

Subjects took 2 capsules with breakfast and 2 capsules with dinner for a 6 wk period. All testing was repeated following 6 wk of supplementation.

### Body Composition

Body composition was assessed by whole body densitometry using air displacement via the Bod Pod^® ^(Life Measurements, Concord, CA). All testing was done in accordance with the manufacturer's instructions as detailed elsewhere [24]. Briefly, subjects were tested wearing only tight fitting clothing (swimsuit or undergarments) and an acrylic swim cap. The subjects wore the exact same clothing for all testing. Thoracic gas volume was estimated for all subjects using a predictive equation integral to the Bod Pod^® ^software. The calculated value for body density was used in the Siri equation [25] to estimate body composition. A complete body composition measurement was performed twice, and if the body fat % was within 0.05% the two tests were averaged. If the two tests were not within 0.05% agreement, a third test was performed and the average of 3 complete trials was used for all body composition variables. All testing was completed first thing in the morning following a 10 h overnight fast (water intake was allowed).

### Resting Metabolic Rate (n = 24)

For logistical reasons, metabolic testing was only performed on the first twelve subjects from each group (n = 24). Subjects refrained from caffeine consumption and vigorous exercise for 24 h prior to the resting metabolic rate (RMR) test. The subjects kept a detailed record of their food intake for the day prior to testing, and this was used to duplicate the diet for the day prior to all subsequent tests. Subjects transported themselves to the lab with the provision that they did not walk more than 100 meters total for their commute. Subjects rested in the supine position in a darkened room covered with a light blanket. A rubber face mask was used to collect expired gases for analysis via open circuit indirect calorimetry using a Medgraphics Ultima Cardio II breath-by-breath system that was calibrated prior to each test according to manufacturers specifications. (Medical Graphics Corporation, St. Paul, MN, USA). While the subjects rested quietly, data were collected for 40 min. The final 20 min of data collected was averaged and 24 h energy expenditure was calculated using the thermal equivalent of O_2 _consumed based on a non-protein RQ table [26].

### Salivary analysis

Subjects rinsed their mouth with water prior to all saliva collections to minimize contamination of the samples. Saliva was collected in a polypropylene vial via passive drool through a short straw and stored at -80°C until analysis. Prior to analysis, samples were thawed and centrifuged at 10,000 g for 20 minutes to remove mucins and analyzed for cortisol concentration using a commercially available enzyme immunoassay kit (Salimetrics, State College, PA, USA). Salivary cortisol is a sensitive marker of activation the hypothalamus-pituitary-adrenal system's response to stress and correlates very well with blood cortisol concentrations [27].

### Statistical Analysis

Data were analyzed using the Statistical Package for the Social Sciences version 13 (SPSS Inc., Chicago, IL). A treatment by time, repeated measures ANOVA was used to evaluate significant differences, and a standard pearson's r was used to evaluate correlations. For all analysis, the alpha level was set at p ≤ 0.05.

## Results

A total of 47 individuals volunteered to participate in this study. Two individuals withdrew from the study citing personal time conflicts, and one participant withdrew from the study as a result of a possible reaction to the safflower oil capsules. In general, both treatments were very well tolerated and no other side effects were noted for either group. Of particular importance, the enteric coating of the fish oil capsules prevented "fish burps," which are a common side effect often experienced with fish oil supplementation. A total of 44 subjects completed the study (Table 1).

### Body Composition

**Figure 1:**
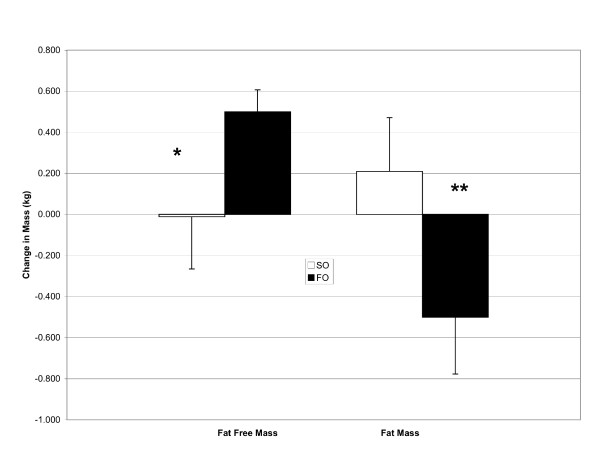
**Change in fat mass and fat free mass following 6 wk of treatment with either 4 g/d of safflower oil (SO), or 4 g/d of fish oil (FO). Data are means ± SEM**. * significant treatment X time interaction, p = 0.04. ** significant treatment X time interaction, p = 0.03

Results from the body composition testing are presented in Table 1. There were no significant differences observed for body mass between the treatments (SO = 0.2 ± 0.8 kg; FO = 0.0 ± 0.9 kg; p = 0.52). However, there was a significant treatment by time interaction observed for fat free mass which means the change in fat free mass over time was significantly different between the treatments (Figure 1: SO = -0.1 ± 1.2 kg; FO = +0.5 ± 0.5 kg; p = 0.03). Similarly, there was a significant treatment by time interaction for fat mass as well (Figure 1: SO = 0.2 ± 1.2 kg; FO = -0.5 ± 1.3 kg; p = 0.04). Percent body fat also tended to change differently over time between the treatments (SO = 0.3 ± 1.5%; FO = -0.4 ± 1.3%; p = 0.08).

### Salivary Cortisol Concentrations

There was a tendency for salivary cortisol concentrations to change differently over time between the two treatments (SO = 0.016 ± 0.272 μg/dL; FO = -0.072 ± 0.142 μg/dL; p = 0.11). However, when a repeated measures t test was performed on the Pre and Post scores of each group independently, the SO change was not significant (p = 0.79), but the Post score was significantly lower than the Pre score in the FO group (p = 0.04). It is very likely that the reduced statistical power of the omnibus F used in the repeated measures ANOVA resulted in a type II error, and the reduction in salivary cortisol concentrations following fish oil supplementation is a real effect. In support of this, the 95% confidence interval of the Pre- Post difference in salivary cortisol concentration for the fish oil group (table 1) contains only negative values (-0.127 to -0.002 μg/dL), whereas the 95% confidence interval for the safflower oil group is centered around a mean difference value of essentially zero (-0.108 to 0.14 μg/dL). Taken together, these additional statistics suggest that the reduction in salivary cortisol concentration observed in the fish oil group is a real effect.

The change in salivary cortisol concentration in the FO group was significantly correlated with the change in % body fat (r = 0.638, p = 0.001), the change in fat free mass (r = -0.504, p = 0.02) as well as the change in fat mass (r = 0.661, p = 0.001). No significant correlations were observed in the SO group between the change in salivary cortisol concentration and the change in % body fat (r = -0.321; p = 0.17), change in fat free mass (r = 0.007; p = 0.98), or the change in fat mass (r = -0.309; p = 0.19).

### Metabolic Data

No significant differences between groups were observed over time for resting metabolic rate (SO = -62 ± 184 kcal, FO = 17 ± 260 kcal; p = 0.40), or for the respiratory exchange ratio (SO = 0.023 ± 0.54; FO = -0.019 ± 0.85, p = 0.16).

## Discussion

The results of this study showed that 6 weeks of supplemental fish oil significantly increased lean mass, and significantly reduced fat mass in healthy adults. This is in agreement with Couet et al. [21], who observed a significant 0.88 kg reduction in fat mass, and a non-significant 0.20 kg increase in lean mass following 3 weeks of an increased consumption of fish oil. In their study, they added fish oil to the diet, but kept total fat and energy constant between the treatments. In the present study, the fish oil was added on top of an ad libitum diet, with instructions given to the subjects to maintain their normal dietary patterns throughout the study. Similarly, Hill et al [22] found a significant reduction in fat mass following 12 weeks of supplementation with fish oil in overweight subjects. They also observed an increase in lean mass in the fish oil group, however, like the data reported by Couet et al. [21], it did not reach significance. Thorsdottir et al. [23] recently found that supplementation with fish oil, or inclusion of fish in an energy-restricted diet resulted in significantly greater weight loss in young men. Additionally, they found that young men taking the fish oil supplements had a significantly greater reduction in waist circumference compared to the control group, or the group that increased their dietary intake of fish.

Unlike the Couet et al. study [21], we did not observe an increase in RMR, or a decrease in RER following fish oil treatment. The failure to find an increase in RMR following fish oil treatment is hard to explain given the significant increase in lean mass observed in the present study. Several studies have shown that lean mass is the largest determinant of RMR [28-30], and decreasing lean mass decreases RMR [31], while increasing lean mass increases RMR [32]. Therefore, it would be expected that the increase in lean mass would correspond to an increased RMR following fish oil treatment. In the Couet et al. study [21], metabolic data were measured for 45 min following a 90 min rest period. This is a longer time period than the 40 min used in the present study. However, it is doubtful that this methodological difference between the studies contributed to the differing effects observed for RMR and RER values since recent studies have shown that very short rest periods (as little as 5 min) produce reproducible results that correlate extremely well with RMR measures made over much longer time periods [33,34]. It is also unlikely that the use of a subset (n = 24) of the total subject population can explain the failure to observe any metabolic changes since analysis of the 24 subjects found that they responded similar to the entire group in regards to body composition changes. It remains unclear why the increased lean mass observed following fish oil treatment did not correspond to an increase in RMR.

Intuitively it would make sense that if fat mass was reduced, but resting metabolic rate did not change following fish oil treatment, then the amount of calories coming from the oxidation of fatty acids should be increased. However, this was not the case in the present study. Although there was an absolute reduction in the RER following fish oil treatment (which would indicate an increased oxidation of fatty acids), the difference was not statistically significant. While it is possible that a type II error was committed and the reduction in RER was a real effect, it is also possible that the fish oil treatment increased fat oxidation at other times during the day such as during exercise [35], or during the post-prandial period [36].

A potential shortcoming of the present study was not using dietary records to monitor the subjects' intake during the study. Although there are several potential problems with the use of dietary records (for a review of inaccuracies with self-recorded diet records see [37]), they would have provided us with some insight into the dietary habits of the subjects during the study. It therefore remains a possibility that the fish oil supplements resulted in the subjects changing their normal dietary habits. Although increasing dietary fat does not generally cause a decrease in voluntary fat intake [38], it has been shown that fish oil may reduce appetite [39], which could have led to the subjects consuming less total calories during the study. While a reduction in volitional food intake would explain the observed reduction in fat mass following fish oil treatment, it does not explain the increase in lean mass we observed.

Although other studies have observed a significant [3,5], or insignificant [21,22], increase in lean mass following fish oil treatment, to date no study has determined the mechanism by which dietary fish oil causes an increased accretion of lean mass. One possibility lies in the well-documented ability of dietary omega 3 fatty acids to reduce inflammatory cytokines [40], since inflammatory cytokines have the ability to increase protein degradation mainly by activating the ATP-ubiquitin-dependent pathway [41-45]. It is possible then, that dietary fish oil is simply decreasing the breakdown of protein tissue caused by inflammatory cytokines, and this results in an increased accretion of protein over time.

An alternative possibility is that fish oil supplementation was able to increase lean mass by reducing cortisol levels since it is well established that cortisol increases protein catabolism [46-49]. The significant negative correlation (r = -0.504, p = 0.02) observed in the fish oil group between the change in lean mass and the change in salivary cortisol concentrations would support this hypothesis. Although other studies have observed a decrease in cortisol levels following fish oil consumption [20], the exact mechanism(s) responsible are currently unknown. However, it is possible that the reduction of IL-6 as a result of fish oil consumption [50] is causing a reduction in cortisol production since it has been shown that IL-6 induces increases in cortisol levels [51,52]. It is unclear whether it is the well-documented ability of fish oil to reduce inflammatory cytokines, the reduction in cortisol, or a combination of both, that resulted in the increased lean mass observed in the present study following fish oil treatment. More work is needed to determine the mechanism(s) responsible for the accretion of lean mass following fish oil consumption.

The role of cortisol in obesity is poorly understood. Excessive cortisol levels, such as those observed in patients with Cushing's disease, results in substantial fat mass gains - especially in the abdominal region [17,19]. However, there is disagreement between studies about the relationship between values of cortisol that are within a normal physiological range, and obesity [18]. Nevertheless, several studies have shown an association with higher levels of cortisol and fat mass [53-58]. In the present study, there was a significant correlation between the change in salivary cortisol and the change in fat mass following fish oil treatment (r = 0.661, p = 0.001). Recent work by Purnell et al. [59] has shown that a reduction in fat mass as a result of dieting does not lower cortisol production, which would suggest that the relationship observed in the present study between salivary cortisol and fat mass was not simply a result of the reduction in fat mass. However, further work is needed to determine exactly how the reduction in cortisol levels may have influenced fat loss observed in the FO group.

In conclusion, 6 weeks of supplemental fish oil significantly increased lean mass, and significantly reduced fat mass in healthy adults. Given the short duration of this study, it is unclear how these changes would impact long-term body composition changes and more research is needed to determine the impact of chronic fish oil supplementation on long-term body composition. The reduction in salivary cortisol following fish oil treatment was significantly correlated with the increased fat free mass and the decreased fat mass observed. To the best of our knowledge, this is the first time that this association has been described in the literature. Since higher salivary cortisol levels are associated with higher mortality rates [60], the reduction in salivary cortisol levels observed in the present study following fish oil supplementation likely has significant implications beyond positive changes in body composition.

## Declaration of Competing interests

The authors declare that they have no competing interests.

## Authors' contributions

EEN was responsible for developing the concept and design of the study, data collection, statistical analysis and manuscript preparation. MJS, MLC, VAP and LKA contributed in the design of the study, data collection, and manuscript preparation. JB contributed with data analysis, statistical analysis, and manuscript preparation. All authors have read and approved the final draft of this manuscript.
